# The uncoating of EV71 in mature late endosomes requires CD-M6PR

**DOI:** 10.1242/bio.059469

**Published:** 2022-09-13

**Authors:** Seii Ohka, Soon Hao Tan, Eri Ishiyama, Katsutoshi Ogasawara, Tomohito Hanasaka, Kinji Ishida, Kyoji Hagiwara, Chia-Chyi Liu, Pele Choi-Sing Chong, Ken-ichi Hanaki, Giampietro Schiavo

**Affiliations:** 1 Neurovirology Project, Tokyo Metropolitan Institute of Medical Science, 156-8506, Tokyo, Japan; 2 Department of Pathology, University of Malaya, 59100, Kuala Lumpur, Malaysia; 3 Technical Support Center for Life Science Research, Iwate Medical University, 028-3694, Iwate, Japan; 4 Vaccine R&D center, National Institute of Infectious Diseases and Vaccinology, National Health Research Institutes, Zhunan Town, Miaoli County, 35053, Taiwan; 5 Molecular NeuroPathobiology Laboratory, Queen Square Institute of Neurology, University College London, London, WC1N 3BG, United Kingdom; 6 UK DRI Group, UK Dementia Research Institute at UCL, London, WC1N 3AR, United Kingdom; 7 UCL Queen Square Motor Neuron Disease Centre, University College London, London WC1N 3BG, United Kingdom

**Keywords:** EV71, Late endosomes, Mannose 6-phosphate receptor, Uncoating, SCARB2

## Abstract

Enterovirus 71 (EV71) is one of the causative agents of hand-foot-and-mouth disease, which in some circumstances could lead to severe neurological diseases. Despite of its importance for human health, little is known about the early stages of EV71 infection. EV71 starts uncoating with its receptor, human scavenger receptor B2 (hSCARB2), at low pH. We show that EV71 was not targeted to lysosomes in human rhabdomyosarcoma cells overexpressing hSCARB2 and that the autophagic pathway is not essential for EV71 productive uncoating. Instead, EV71 was efficiently uncoated 30 min after infection in late endosomes (LEs) containing hSCARB2, mannose-6-phosphate receptor (M6PR), RAB9, bis(monoacylglycero)phosphate and lysosomal associated membrane protein 2 (LAMP2). Furthering the notion that mature LEs are crucial for EV71 uncoating, cation-dependent (CD)-M6PR knockdown impairs EV71 infection. Since hSCARB2 interacts with cation-independent (CI)-M6PR through M6P-binding sites and CD-M6PR also harbor a M6P-binding site, CD-M6PR is likely to play important roles in EV71 uncoating in LEs.

## Abstract (precis)

**Summary:** In human rhabdomyosarcoma cells, EV71 uncoating takes place in mature late endosomes and relies on CD-M6PR.

## INTRODUCTION

Enterovirus 71 (EV71) is a causative agent of hand-foot-and-mouth disease (HFMD) in young children and infants (Hagiwara et al., 1978; Blomberg et al., 1974), yet infection with this virus occasionally leads to severe neurological diseases, such as brainstem encephalitis and acute flaccid paralysis (McMinn, 2002). EV71 is classified into Enterovirus within the family Picornaviridae (Racaniello, 2007), which contains positive-sense single-stranded RNA surrounded by icosahedral capsid proteins. Sixty copies of the four structural proteins VP1, VP2, VP3, and VP4 assemble to form the capsid (Plevka et al., 2012; Rossmann and Johnson, 1989; Wang et al., 2012). Functional EV71 receptor, human scavenger receptor class B, member 2 (hSCARB2, also known as lysosomal integral membrane protein II or CD36b like-2) (Yamayoshi et al., 2009), interacts with residues located within an indentation of the viral capsid, called the canyon (Dang et al., 2014). After the binding of hSCARB2 to the EV71 capsid, the virus particle releases viral genomic RNA using VP1 and VP4 under acidic conditions (Yamayoshi et al., 2013; Dang et al., 2014).

hSCARB2, a CD36 family member containing two transmembrane domains (Vega et al., 1991), is an intrinsic receptor for β-glucocerebrosidase (β-GC) (Eskelinen et al., 2003). It transports β-GC from the endoplasmic reticulum (ER) to lysosomes (Blanz et al., 2010; Reczek et al., 2007), and is required for lysosomal integrity (Eskelinen et al., 2003). In primate cells, lysosome sorting of β-GC by hSCARB2 is aided by cation-independent (CI)-mannose-6-phosphate (M6P) receptor (M6PR) (Zhao et al., 2014), which binds to a M6P group linked to amino acid 325 of hSCARB2; however, cation-dependent (CD)-M6PR may also contribute to the lysosome sorting of β-GC by hSCARB2 (Zhao et al., 2014). β-GC and EV71 bind overlapping regions of the intraluminal domain of hSCARB2: β-GC interacts with the 145-222 portion of hSCARB2 (in particular 150-167) (Zachos et al., 2012; Reczek et al., 2007), whereas amino acids 142-204 (and in particular 144-151) are crucial for EV71 binding (Chen et al., 2012; Yamayoshi and Koike, 2011).

It has been reported that small interfering RNAs (siRNAs) against clathrin heavy chain inhibit EV71 infection, suggesting that EV71 exploits a clathrin-dependent pathway for its entry into target cells (Lin et al., 2012; Hussain et al., 2011). After internalization, EV71 is delivered to early endosomes (Yamayoshi et al., 2013), however, the precise mechanism for EV71 uncoating, such as its timing and localization, has not been clarified. In the endocytic pathway, early endosomes mature to late endosomes (LEs), which then fuse with lysosomes containing lysosomal associated membrane protein (LAMP)-1 or 2. Progression along the endosomal route is regulated by specific RAB GTPases, which serve as specific markers of these organelles. RAB5 regulates the early steps in the endocytic pathway, participating to clathrin coated vesicle uncoating and formation of early endosomes (Semerdjieva et al., 2008; Pfeffer, 2017), whilst RAB7 regulates the transition between early endosomes and LEs (Wandinger-Ness and Zerial, 2014), and RAB9 functions in LEs and the trans-Golgi network (Pfeffer, 2017).

LAMP-1 and LAMP-2 are lysosomal membrane proteins, which share common functions *in vivo*. Beyond their roles in maintaining the structural integrity of lysosomes, both LAMPs are involved in the regulation of autophagic vacuoles and unesterified cholesterol in embryonic fibroblasts (Eskelinen, 2006). In previous studies, SCARB2 was found to co-localize with LAMP-2 in primate cells (Zachos et al., 2012).

Here, we report that EV71 is efficiently uncoated 30 min after infection (mai) in mature LEs. CD-M6PR was found to be essential for EV71 replication by siRNA knockdown, suggesting that CD-M6PR may play an important role at early stages of EV71 infection.

## RESULTS

### Analysis of EV71 uncoating kinetics

**Fig. 1.**
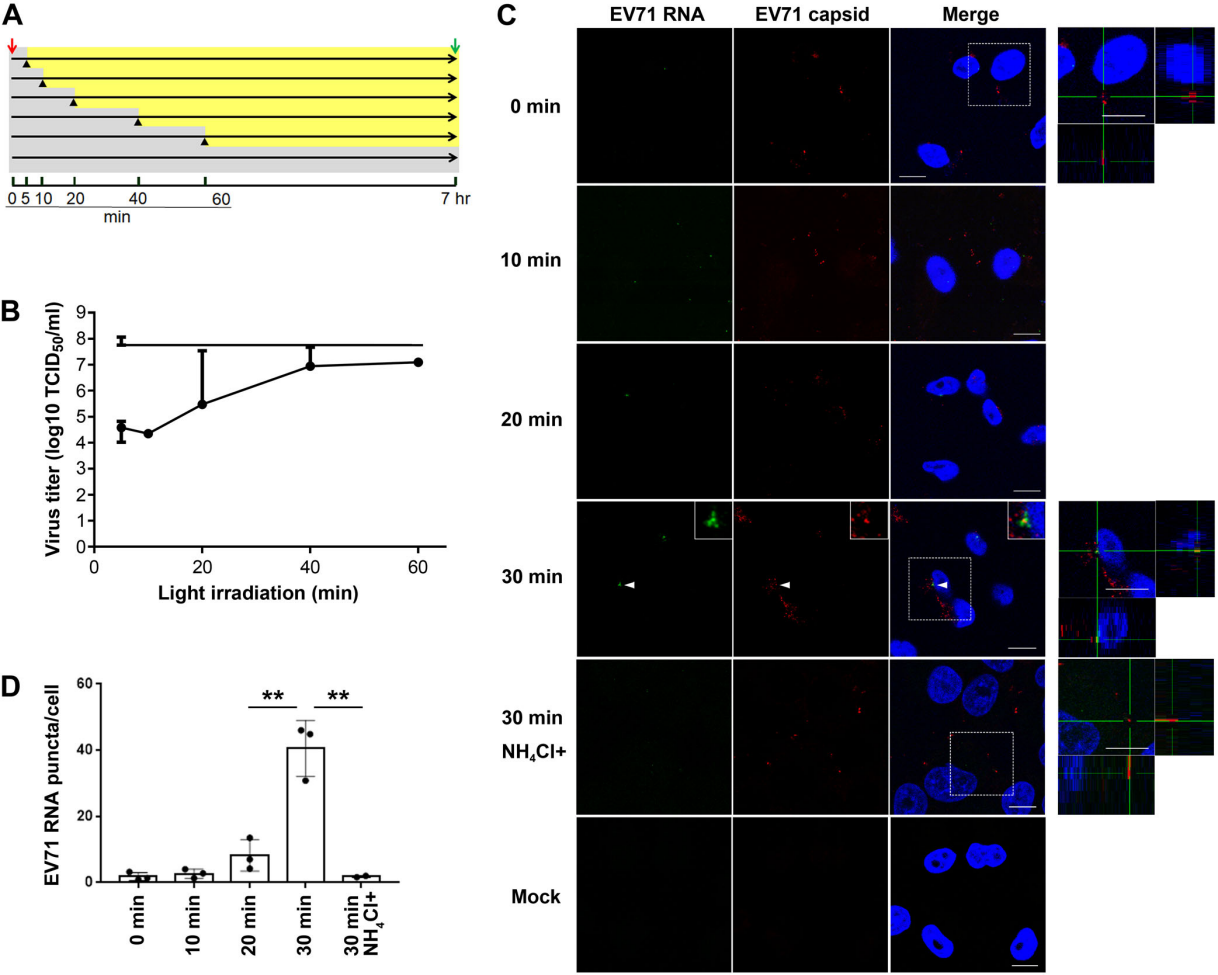
**EV71 is uncoated at 30 mai and is dependent on acidification.** (A) Experimental protocol for infection of RD-hSCARB2 cells with light-sensitive EV71. The red arrow indicates virus addition, the green arrow indicates the recovery of the infected cells followed by the titration of the live virus, black arrowheads indicate the start of light irradiation, gray areas indicate the periods without the light irradiation, and yellow area indicates the periods with the light irradiation. The time after the infection with light-sensitive virus is indicated. (B) Virus titer of the recovered virus. Horizontal axis indicates the onset of light irradiation after infection. The top line represents the virus titer without irradiation. Error bars represent s.d. (C) *In situ* hybridization for RNA genome of EV71 and immunofluorescence for the capsid proteins after infection of RD-hSCARB2 cells with EV71 in the absence or presence of NH_4_Cl. The time of fixation after infection is indicated. DAPI is in blue, EV71 RNA genome is in green, whilst EV71 capsid antigens are in red. The panels on the right are enlarged 3D cross-section views of the dashed rectangles in the merged panels. Arrowheads indicate EV71 RNA+capsid double-labelled clustered vesicles. Insets are 400% enlarged panels of the arrowhead areas. Representative images are shown. Scale bars: 10 µm. (D) The numbers of visible green dots corresponding to EV71 RNA genomes per cell were shown. Number of z stack sets were three except for the 30 min NH_4_Cl+ sample (two). **P<0.01. Statistical significance was determined by a two-tailed, unpaired *t*-test. Error bars represent s.d.

EV71 is uncoated upon binding to hSCARB2 at low pH (Yamayoshi et al., 2013). However, it is unclear where and when uncoating occurs in cells. To clarify when EV71 starts uncoating, we used light-sensitive EV71, which can be inactivated after irradiation at 450 nm. Human rhabdomyosarcoma (RD) cells overexpressing hSCARB2 (RD-hSCARB2), were infected with light-sensitive EV71 at a multiplicity of infection (MOI) of ten and cells were either kept in the dark or irradiated at the indicated time after infection to inactivate intact virus. Seven hours after infection, cells were harvested and the viral titers were determined (Fig. 1A). In samples irradiated at either 5 or 10 mai, virus titers decreased by 3 logs as compared to samples kept in the dark (Fig. 1A and 1B). In contrast, the viral titer of the sample irradiated at 20 mai was found to increase as compared to that in the 10 mai sample, and a further increase of viral titer was observed at 40 mai (Fig. 1A,B). The viral titer was found to plateau at 60 mai, suggesting that the uncoating of EV71 is likely to start after 10 mai and that all the productive viral particles were uncoated before 40 mai. The experiments were repeated three times and similar results were observed.

Next, we combined *in situ* hybridization and immunofluorescence to visualize the viral RNA as well as the viral capsid. RD-hSCARB2 cells were infected with purified EV71 at an MOI of 25 and cells were fixed at specific times after infection, followed by immunofluorescence and *in situ* hybridization (Fig. 1C). The number of EV71 genomic RNA puncta per cell were analyzed (Fig. 1D); we found 1.4, 0.9 and 3.2 for 0 mai, 2.8, 4.0 and 1.3 for 10 mai, 4.2, 13.5 and 7.0 for 20 mai, 46.0, 30.8 and 44.9 for 30 mai, 1.6 and 2.0 for 30 mai with NH_4_Cl. At 0 mai, we observed negligeable amounts of EV71 genomic RNA (1.8 puncta per cell), whereas we detected EV71 capsid on the cell surface. This result suggests that EV71 genomic RNA inside the capsid cannot be detected by our method. At 10 mai, we detected EV71 capsid, but we observed only 2.7 puncta of EV71 genomic RNA per cell, indicating that EV71 was not uncoated efficiently at this time point. At 20 mai, we observed 8.2 puncta of EV71 genomic RNA per cell, whilst at 30 mai, 40.6 puncta of EV71 genomic RNA per cell were observed. The amount of EV71 genomic RNA significantly increased around five times at 30 mai compared to that at 20 mai. Furthermore, EV71 RNA and capsid double-labelled vesicles, and EV71 RNA abutting capsid-positive structures underwent clustering at 30 mai, suggesting that uncoating of EV71 robustly occurs at this time point (*n*=2 independent experiments).

To confirm whether acidification is required for uncoating, cells were treated with NH_4_Cl, which blocks acidification of the lumen of endocytic organelles, and the presence of EV71 genomic RNA was then assessed. RD-hSCARB2 cells were pretreated with or without 40 mM NH_4_Cl for 30 min followed by addition of EV71 at an MOI of 25. As shown in Fig. 1C, we observed only 1.8 puncta of EV71 genomic RNA per cell even at 30 mai upon incubation with NH_4_Cl, demonstrating that an acidic pH is absolutely required for EV71 uncoating. These experiments were repeated three times. Our results confirm previous finding that EV71 uncoating is triggered by low pH (Yamayoshi et al., 2013; Chen et al., 2012), and suggest that this immunofluorescence approach is a reliable approach for the detection of viral uncoating.

### EV71 localizes to LEs during uncoating

**Fig. 2.**
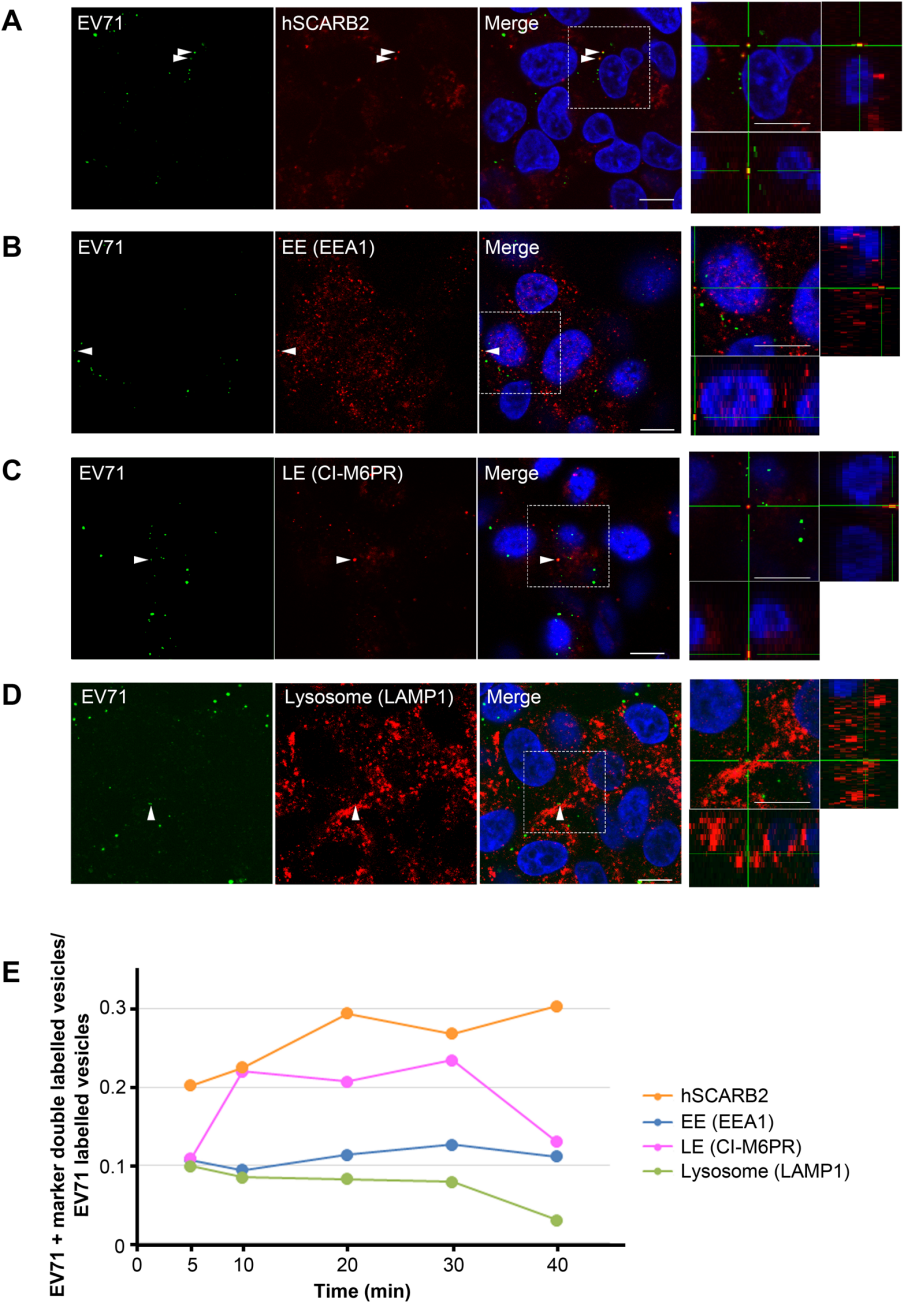
**EV71 co-distributes with hSCARB2 and M6PR, but not with LAMP1 until 40 mai.** Co-localization of EV71 and hSCARB2 (A), EEA1 (B), M6PR (C), or LAMP1 is shown by immunofluorescence (D). RD-hSCARB2 cells were infected with EV71 and fixed 30 min (B-D) or 40 min (A) after infection followed by immunofluorescence. DAPI is in blue, EV71 antigen is in green, whereas hSCARB2 or other markers are in red. The panels on the right are enlarged 3D cross-section views of the dashed rectangles in the merged panels. Arrowheads indicate co-localization of EV71 with markers. Representative images are shown. Scale bars: 10 µm. (E) Kinetics of co-localization of EV71 with hSCARB2 or other markers. Times indicate the time after infection.

It has been hypothesized that EV71 requires hSCARB2 for its uncoating (Yamayoshi et al., 2013). To begin with, we examined whether EV71 traffics with hSCARB2 during the uncoating period using immunofluorescence (Fig. 2). RD-hSCARB2 cells were infected with EV71 at an MOI of 25, and fixed at 5, 10, 20, 30, and 40 mai followed by immunofluorescence. We detected 129, 111, 102, 123, and 102 EV71-positive puncta at 5, 10, 20, 30, and 40 mai, respectively. As shown in Fig. 2A and E (Fig. 2A for 40 mai; immunofluorescence at 5, 10, 20, 30 mai not shown; Fig. 2E, co-localization kinetics), EV71 displays a high level of co-localization with hSCARB2 ranging from 20 to 30% until 40 mai, suggesting that EV71 is sorted together with hSCARB2 in the uncoating period. Next, we examined the intracellular localization of EV71 during this period. As stated in the Materials and Methods, anti-early endosome antigen 1 (EEA1) antibodies were used to identify early endosomes (EE), an anti-CI-M6PR/IGF2R antibody was used for LEs and anti-LAMP1 antibodies were used as lysosome marker. We analyzed a large number of EV71-positive puncta: 60, 92, and 81 at 5 mai, 102, 112, and 100 at 10 mai, 121, 112, and 93 at 20 mai, 103, 101, and 101 at 30 mai, 100, 101, and 103 at 40 mai, for EEA1, CI-M6PR, and LAMP1, respectively. Interestingly, EV71 were found to co-localize with CI-M6PR in around 20% of the EV71-positive organelles from 10 to 30 mai (Fig. 2C for 30 mai; immunofluorescence data at 5, 10, 20, 40 mai not shown; Fig. 2E, co-localization kinetics), whereas only 10% of EV71 co-localized with EEA1 or LAMP1 puncta at this time point (Fig. 2B and 2D for 30 mai; immunofluorescence at 5, 10, 20, 40 mai not shown; Fig. 2E, co-localization kinetics; *n*=3 independent experiments). We confirmed that all EV71 puncta positive for the above markers also co-localize with hSCARB2.

**Fig. 3.**
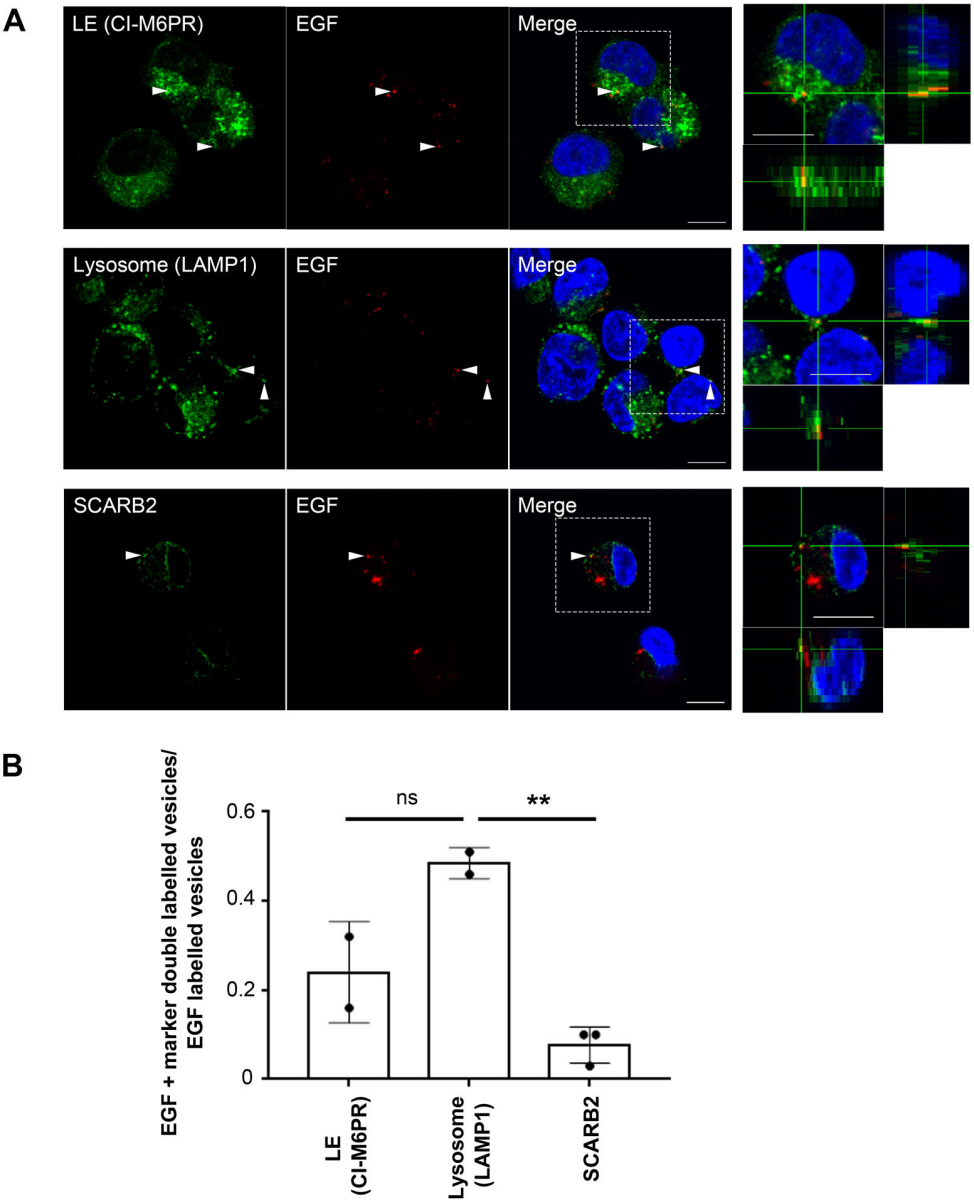
**EGF co-localizes with LAMP1 at 30 mai.** (A) Co-localization of EGF with M6PR, LAMP1 or SCARB2 is shown by immunofluorescence of EGF and M6PR, LAMP1 and SCARB2. RD-hSCARB2 cells were infected with EV71 and then fixed 30 min after the addition of EGF followed by immunofluorescence. DAPI is in blue, EGF is in green, whereas organelle markers are in red. The panels on the right are enlarged 3D cross-section views of the dashed rectangles in the merged panels. Arrowheads indicate co-localization of EGF with markers. Representative images are shown. Scale bar: 10 µm. (B) Quantification of co-localization of EGF with specific markers. Number of z stack sets was two except SCARB2 was three. ns, not significant, **P<0.01. Statistical significance was determined by a two-tailed, unpaired *t*-test. Error bars represent s.d.

As a control, we checked the intracellular localization of epidermal growth factor (EGF), which is known to traffic to LAMP1-positive LE/lysosomes 30 min after internalization (Fig. 3) (Chin et al., 2020). As expected, EGF co-localized with LAMP1 in 48.5% of the organelles, whereas it co-distributes with CI-M6PR only in 24.0% of the puncta (50 EGF+CI-M6PR double-labelled vesicles in 253 EGF-positive puncta; 123 EGF+LAMP1 double-labelled vesicles in 261 EGF-positive puncta; 9 EGF+SCARB2 double-labelled vesicles in 117 EGF-positive puncta). These results suggest that EGF is targeted to lysosomes 30 min after internalization in our experimental conditions (*n*=2 independent experiments).

**Fig. 4.**
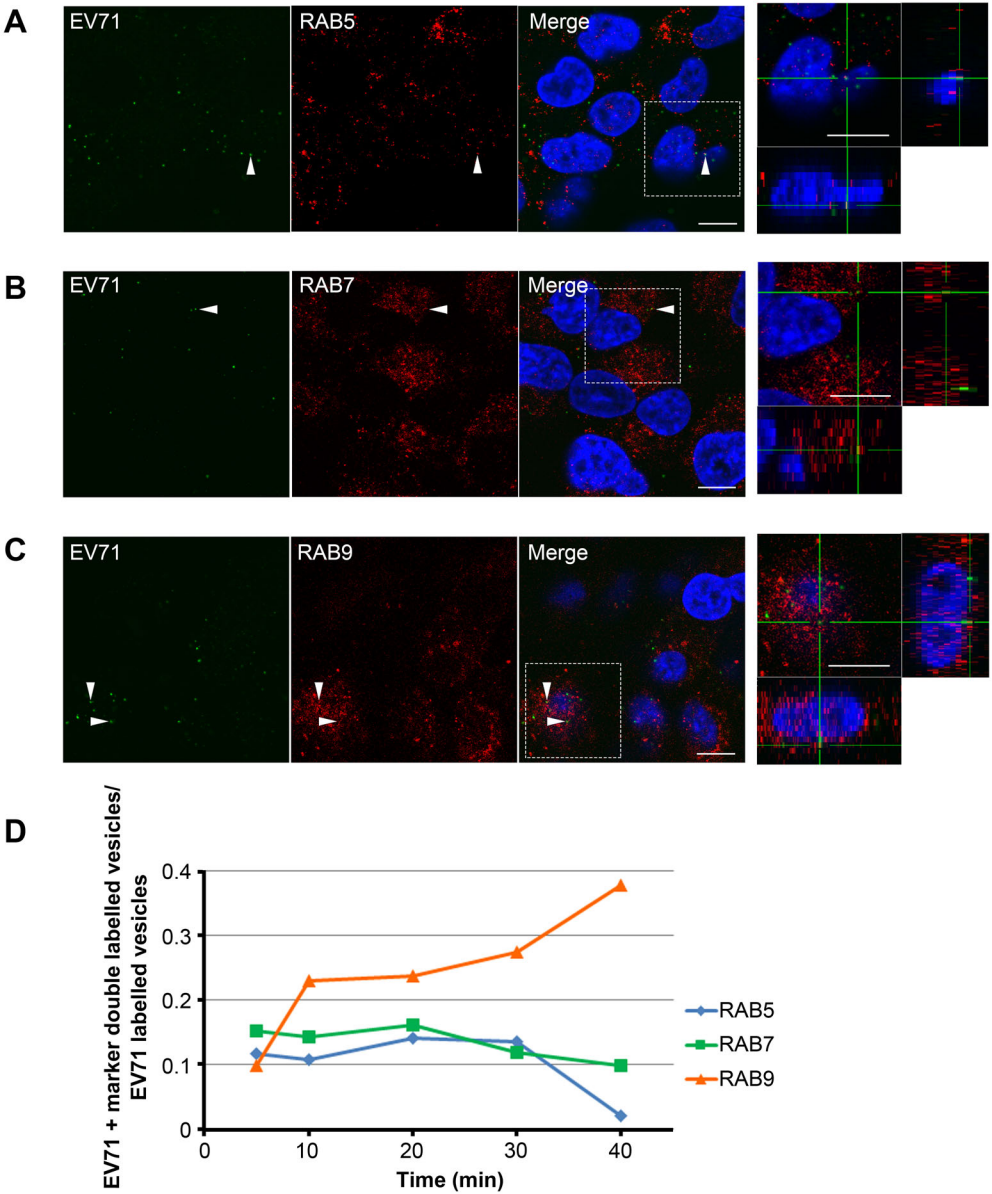
**EV71 co-distributes with RAB9 during the uncoating period.** Co-localization of EV71 with RAB5, RAB7, or RAB9 is shown by immunofluorescence of EV71 and RAB5 (A), RAB7 (B), or RAB9 (C). RD-hSCARB2 cells were infected with EV71 and fixed 30 min after infection, followed by immunofluorescence. DAPI is in blue, EV71 antigen is in green, whereas marker antigens are in red. The panels on the right are enlarged 3D cross-section views of the dashed rectangles in the merged panels. Arrowheads indicate co-localization of EV71 with markers. Representative images are shown. Scale bars: 10 µm. (D) Kinetics of co-localization of EV71 with the different markers. Times indicate the time after infection.

To confirm EV71 localization during uncoating, we tested the co-distribution of EV71 with the endosomal markers RAB5, RAB7, and RAB9. RAB5 mainly localizes on early endosomes, whereas RAB7 and RAB9 localizes on specific sub-classes of LEs. RD-hSCARB2 cells were infected with EV71 at an MOI of 25, and fixed at 5, 10, 20, 30, and 40 mai followed by immunofluorescence. We analyzed the following number of EV71-positive puncta; 153, 135, and 128 at 5 mai, 182, 143, and 150 at 10 mai, 145, 154, and 137 at 20 mai, 133, 174, and 150 at 30 mai, 35, 117, and 102 at 40 mai for RAB5, RAB7, and RAB9, respectively. EV71 shows an extensively colocalization with RAB9 from 10 mai in over 20% of the organelles (Fig. 4C,D), whereas the co-distribution with RAB5 or RAB7 occurs in less than 20% of the cases during this period (Fig. 4A,B,D; data at 5, 10, 20, 40 mai are not shown; *n*=3 independent experiments). These results indicate that EV71 enters RAB9-positive LE during the uncoating period.

The data shown in Fig. 2 suggest that EV71 is not targeted to LAMP1-positive endolysosomes. Since it has been reported that hSCARB2 co-localized with LAMP2 (Reczek et al., 2007), EV71 may be targeted to LAMP2-positive organelles during uncoating. To investigate whether this is the case, RD-hSCARB2 cells were infected with EV71 at an MOI of 25, and fixed at 30 and 40 mai, followed by immunofluorescence (Fig. S1A,C). Around 20% of EV71 were found to co-localize with LAMP2 at 30 mai, whereas the co-localization decreases around 5% at 40 mai. The experiments were repeated three times and the same trend was observed. These results suggest that the organelles containing EV71 are positive for LAMP2 at 30 mai, but LAMP2 is sorted away before 40 mai. Altogether, our findings demonstrate that EV71 is targeted to endocytic organelles containing LAMP2, RAB9, and CI-M6PR at 30 mai prior to uncoating.

### Uncoating of EV71 does not require LAMP2

It has been previously reported that a specific pool of autophagosomes contains LAMP2, RAB9, and M6PR (Nishida et al., 2009) and that 3-methyladenine (3-MA), which inhibits formation of amphisomes (Nishida et al., 2009; Juenemann and Reits, 2012; Sun et al., 2015), halts immature LEs to gain LAMP2. To examine whether blocking autophagy affects the co-localization of EV71 with LAMP2, RAB9, and M6PR at 30 and 40 mai, cells were treated with 3-MA and organelles containing EV71 were analyzed by immunofluorescence and quantified (Fig. S1B,C). RD-hSCARB2 cells were pretreated with or without 5 mM 3-MA for 30 min followed by addition of EV71 at an MOI of 25. We analyzed the following numbers of EV71-positive puncta; 106, 130, and 140 at 30 mai without 3-MA, 129, 120, and 202 at 30 mai with 3-MA, 132, 181, and 125 at 40 mai without 3-MA, 103, 145, and 109 at 40 mai with 3-MA, for LAMP2, RAB9, and CI-M6PR, respectively. In the presence of 3-MA, EV71 was not targeted to an intracellular compartment positive for LAMP2 even at 40 mai. Interestingly, 3-MA did not affect the ability of EV71 to reach organelles containing RAB9 or CI-M6PR at 30 and 40 mai. The experiments were repeated three times with similar results. These findings suggest that 3-MA blocks the targeting of EV71 to LAMP2-positive organelles yet does not alter its co-localization with RAB9 or CI-M6PR.

To investigate whether LAMP2 is necessary for EV71 uncoating, the effect of 3-MA on light-sensitive EV71 was examined (Fig. S2A). RD-hSCARB2 cells were infected with light-sensitive EV71 at an MOI of 0.004 in the presence or absence of 5 mM 3-MA. Cells were either kept in the dark or irradiated at 450 nm at 10 or 40 mai to inactivate the intact virus. After irradiation, in selected samples, 3-MA-containing medium was replaced with fresh medium to assess the inhibitory effect this compound on viral replication. Twenty-three hours after infection, cells were harvested, and the viral titers determined.

In the light-treated samples, viral uncoating was blocked by irradiation, hence the effect of viral uncoating on the viral replication was restricted to the pre-irradiation period. When cells were irradiated at 10 mai, the virus titer decreased by around 5 logs compared to cells kept in the dark in the absence of 3-MA, indicating that this genetically-engineered virus was indeed very sensitive to light. First, we investigated the effect of 3-MA on the productive uncoating of the virus. Light irradiation inhibits additional viral uncoating to enable solely observing the productive uncoating before light irradiation. With light irradiation, 3-MA significantly decreased viral titer in 3-MA (+) to (−) and in 3-MA (+) (*P*=0.0118 and *P*=0.0057, respectively; unpaired *t*-test) compared to 3-MA (−). These results indicate that 3-MA inhibited viral productive uncoating before light irradiation, although the virus titer decreased only less than one order of magnitude. Next, we investigated the effect of 3-MA on the replication of the virus. Treatment with 3-MA during viral replication after light irradiation [3-MA (+)] reveals the effect of autophagy on the viral replication process. 3-MA did not decrease viral titer in 3-MA (+) compared to 3-MA (+) to (−) (*P*>0.05 by unpaired *t*-test), suggesting that 3-MA did not have a significant effect on the viral replication after light irradiation. No significant differences were found in samples kept in the dark, indicating the viral uncoating progressed after 40 mai in absence of illumination. The experiments were repeated three times and similar results were observed. These findings suggest that 3-MA has only a limited effect on EV71 productive uncoating, and that this process does not require transit through LAMP2-positive organelles.

To further explore whether EV71 uncoating fully proceed in the presence of 3-MA, we analyzed the influence of 3-MA on viral uncoating by combined *in situ* hybridization and immunofluorescence (Fig. S2B). RD-hSCARB2 cells were infected with EV71 at an MOI of 25 with or without 3-MA, prior to fixing at specific times after infection. At 0 mai, EV71 RNA was not detected, in agreement to the results shown in Fig. 1C, whilst at 30 mai, viral RNA was detected even in the presence of 3-MA. These results confirm that EV71 undergoes uncoating in the presence of 3-MA in a process that does not require LAMP2. To further confirm EV71 undergoes uncoating in LE in the presence of 3-MA, co-localization of EV71 RNA and CI-M6PR was observed (Fig. S2C). At 20 mai, the numbers of EV71 RNA+CI-M6PR double labelled puncta in the absence or presence of 3-MA were 19 and 18, respectively, and the numbers of EV71 RNA labelled puncta in the absence or presence of 3-MA was 106 and 64, respectively. At 30 mai, the EV71 RNA+CI-M6PR double-labelled puncta in the absence or presence of 3-MA were 15 and 48, respectively, whereas the EV71 RNA-labelled puncta in the absence or presence of 3-MA were 167 and 200. Co-localization rate of EV71 RNA and CI-M6PR was significantly higher in the presence of 3-MA compared to that observed in the absence of the compound at 30 mai. A similar trend was seen at 20 mai.

In summary, these results suggest that EV71 undergoes uncoating in LEs containing CI-M6PR even in the presence of 3-MA, and this inhibitor boosts the rate of EV71 uncoating in LEs containing CI-M6PR.

### Uncoating of EV71 occurs in mature LEs containing intraluminal vesicles

**Fig. 5.**
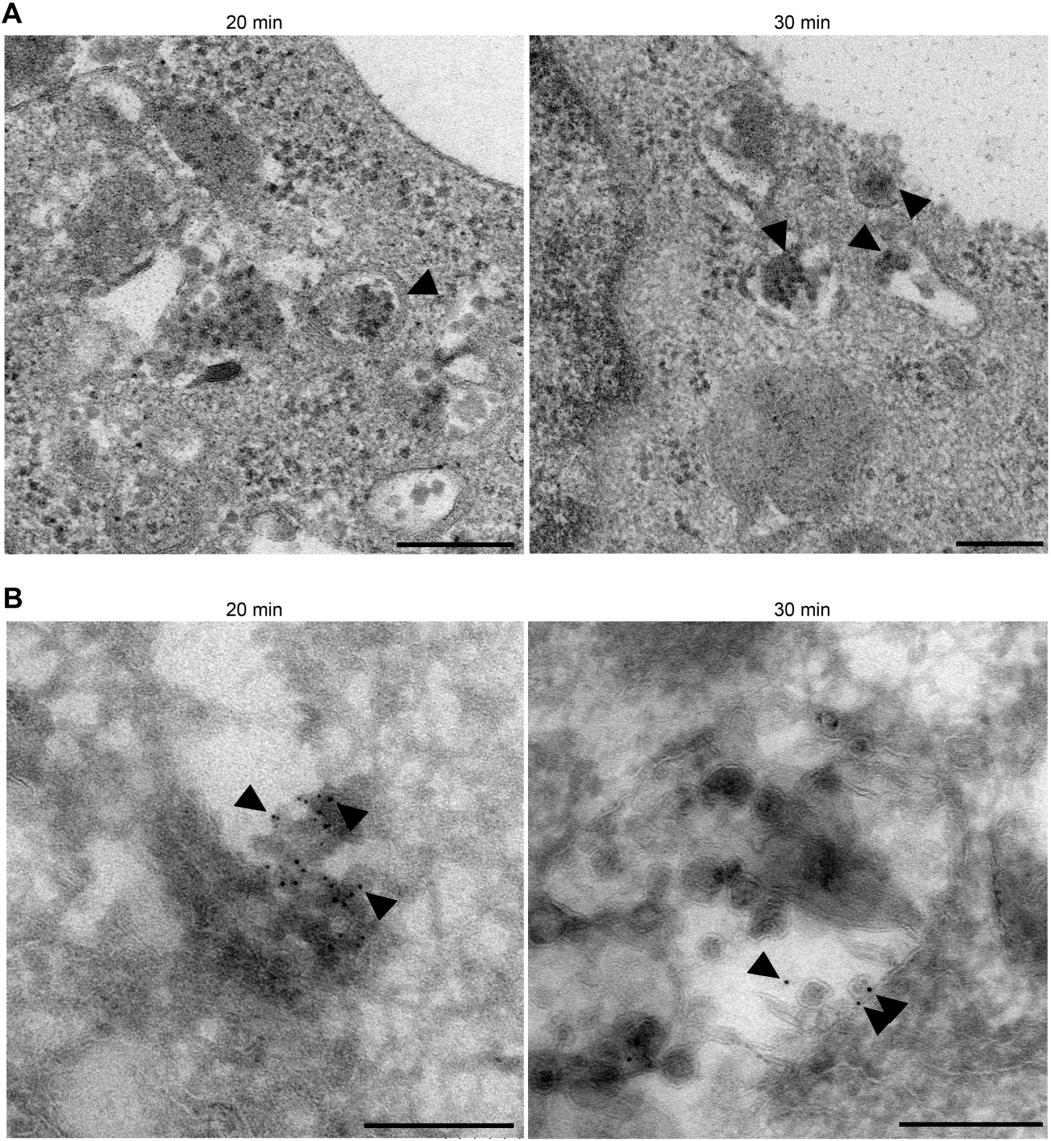
**EV71 particles are incorporated into LEs.** (A) Transmission electron microscopy. RD-hSCARB2 cells were infected with EV71 and fixed at 20 or 30 mai. Times indicate the time after infection. Arrowheads indicate representative EV71-like particles in LE. (B) Immuno-electron microscopy. Gold particles label EV71 antigen. Arrowheads indicate representative gold particles existed in the lumen of single membrane compartments without ILV at 20 mai, and representative gold particles accumulated in single membrane organelles containing ILVs at 30 mai. The experiments were repeated two times and the same trends were observed. Scale bars: 200 nm.

EV71 uncoating starts between 20 mai, when uncoating is negligible, and 30 mai, a time-point in which robust uncoating is observed, yet we lack key information on the nature of these organelles. To further investigate the intracellular trafficking of EV71, we performed transmission electron microscopy (TEM). RD-hSCARB2 cells were infected with purified EV71 at an MOI of 25, fixed at 20 and 30 mai, and then analyzed by TEM (Fig. 5A). At 20 mai, viral particles were surrounded by a single membrane bilayer in organelles resembling LEs, but lacking intraluminal vesicles (ILVs). At 30 mai, the viral particles reach LEs containing in some instances ILVs (*n*=3). These results imply that EV71 is present in the lumen of LEs during its uncoating.

To confirm this conclusion, samples were examined by immunoelectron microscopy (Fig. 5B). At both 20 and 30 mai, gold particles labelling EV71 were found in the lumen of single membrane organelles, likely to be LEs. At 30 mai, gold particles accumulated in single membrane organelles containing ILVs. These results suggest that EV71 accumulates in LE at 20 and 30 mai and that during the uncoating period (30 mai), EV71 specifically localizes at LEs containing ILVs.

**Fig. 6.**
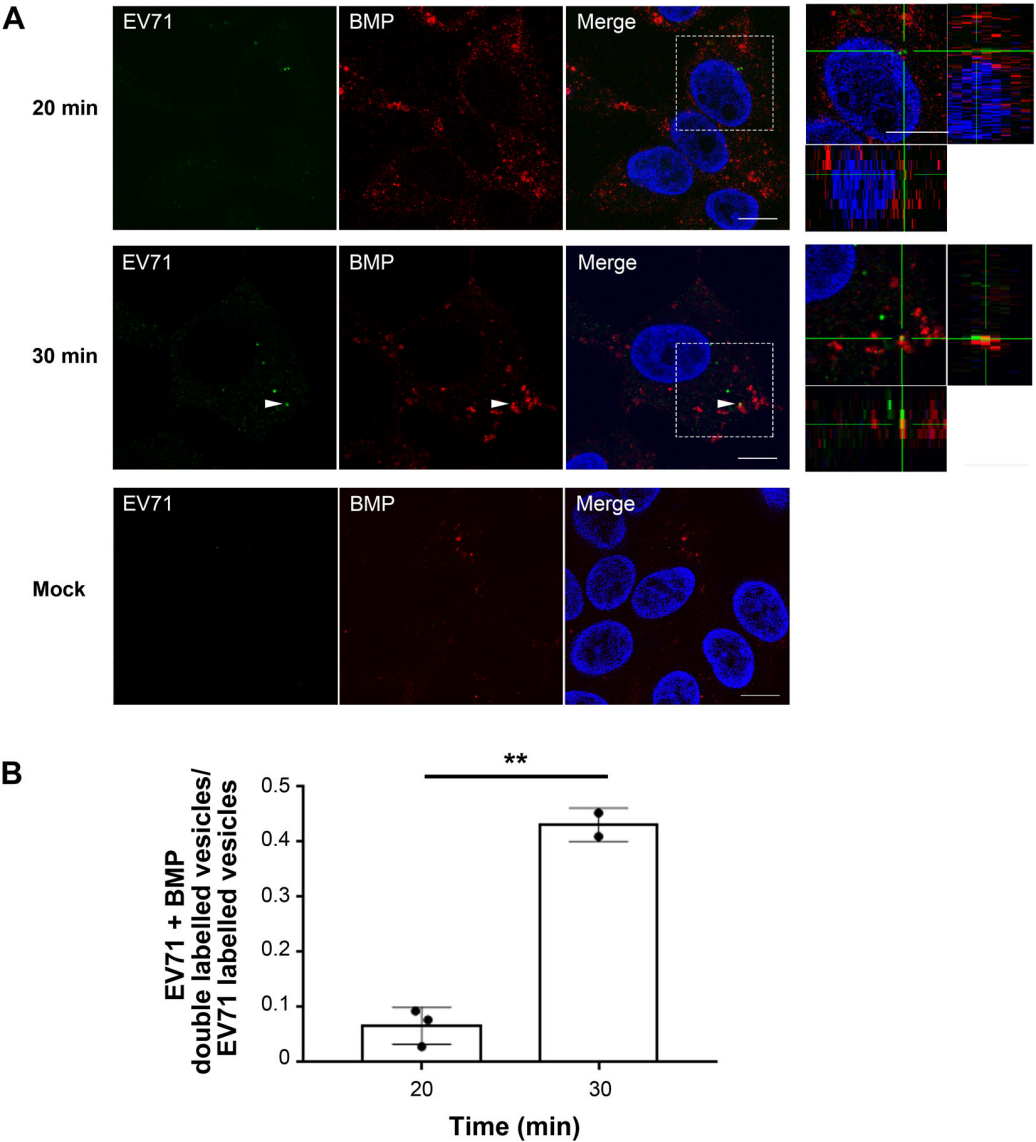
**EV71 translocates into BMP-enriched LE at 30 mai.** (A) RD-hSCARB2 cells were infected with EV71 and then fixed 20 min or 30 min after the infection followed by immunofluorescence. DAPI is in blue, EV71 antigen is in green, whereas BMP is labelled in red. The panels on the right are enlarged 3D cross-section views of the dashed rectangles in the merged panels. Arrowheads indicate co-localization of EV71 with BMP. Representative images are shown. Scale bars: 10 µm. (B) Co-localization rate of EV71 with BMP at 20 or 30 mai. **P<0.01. Statistical significance was determined by a two-tailed, unpaired *t*-test. Error bars represent s.d.

It has been reported that ILVs accumulate in LEs following maturation of this endosomal compartment (Nour and Modis, 2014). ILVs in mature LEs are enriched in M6PRs and bis(monoacylglycero)phosphate (BMP) (Nour and Modis, 2014). Based on these considerations, it is plausible that the organelles containing EV71 at 20 mai are immature LEs, which undergo maturation at 30 mai acquiring ILVs enriched in BMP. To examine whether the BMP content differs between the vesicles containing EV71 at 20 mai and those at 30 mai, RD-hSCARB2 cells were infected with EV71 at an MOI of 25, fixed at 20 and 30 mai and analyzed by immunofluorescence (Fig. 6). We analyzed the following numbers of EV71-positive puncta: 204, 13, 35 at 20 mai and 317, 83 at 30 mai; EV71+BMP double-labelled puncta were 19, 1, 1 at 20 mai and 144, 34 at 30 mai, respectively. At 20 mai, 6.6% of EV71 co-localized with BMP, whereas the percentage of EV71 co-localized with BMP increased to 43.1% at 30 mai (*n*=2 independent experiments), suggesting that LE maturation is important for efficient uncoating of the virus.

### CD-M6PR is required for EV71 replication

**Fig. 7.**
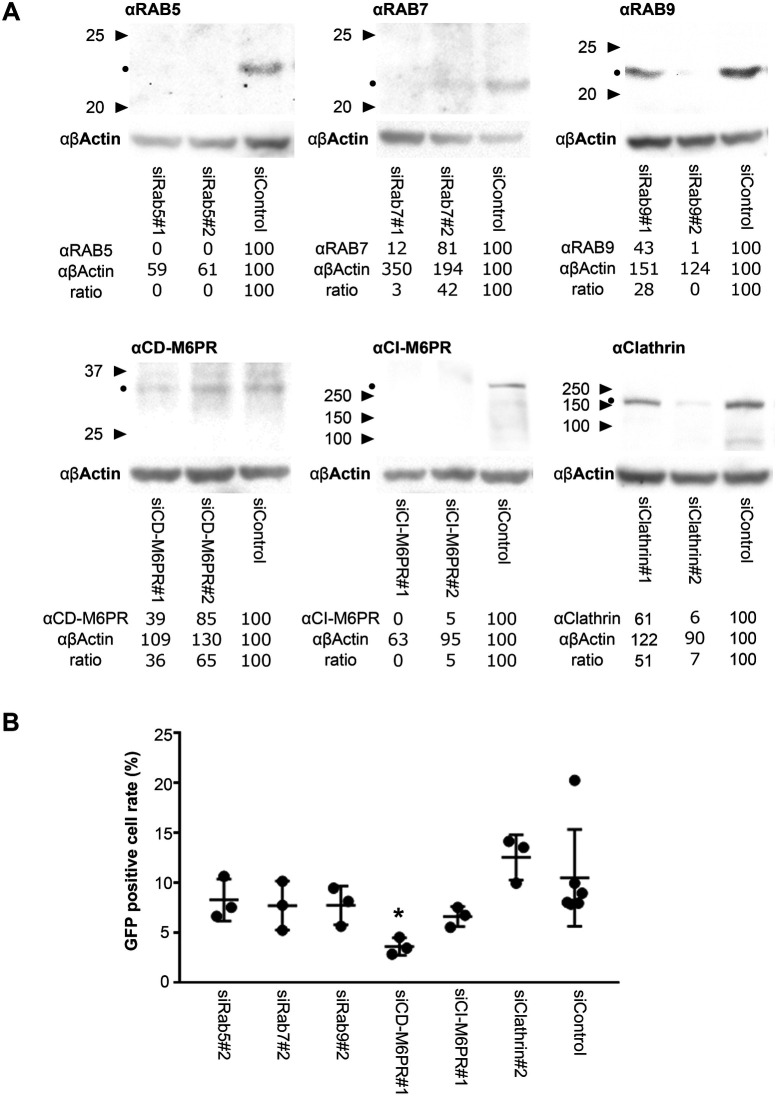
**CD-M6PR is essential for EV71 replication.** (A) Western blotting of RAB5, RAB7, RAB9, CD-M6PR, CI-M6PR and clathrin heavy chain in cells in which these genes have been targeted by siRNA knockdown. The expression of β-actin was examined as loading control. Triangles indicate molecular weight markers. Black dots indicate the predicted molecular weight of the proteins. The numbers are the densities and the ratio of anti-markers/anti β-actin. (B) RD cells were treated with siRNAs targeting RAB5, RAB7, RAB9, CD-M6PR, CI-M6PR or clathrin heavy chain and then infected with EV71-GFP. Cells were fixed at 31 h after infection, followed by fluorescent imaging. GFP-positive cells were counted and their percentage against the total number of DAPI-positive cells plotted. *P<0.05. Statistical significance was determined by a two-tailed, unpaired *t*-test. Error bars represent s.d.

To investigate whether RABs or M6PRs are essential for EV71 trafficking and replication, we analyzed viral replication in cells in which specific components of the endocytic pathway were downregulated by siRNA. RD cells targeted by siRNA knockdown were infected with EV71-GFP at an MOI of 0.5, fixed after 31 h from infection prior to fluorescent imaging to assess the percentage of GFP-positive cells in the culture. Protein expression levels in RD cells treated with siRNA were examined by western blotting (Fig. 7A). As shown in Fig. 7B, CD-M6PR siRNA knockdown showed significant inhibition of EV71 replication compared to cells treated with control siRNA (*P*=0.049), whereas RABs or clathrin heavy chain downregulation did not have any effect (*n*=3 independent experiments). These results suggest that RAB5, RAB7, and RAB9 are not essential for EV71 replication, whereas CD-M6PR is required for this process, including virus uncoating.

## DISCUSSION

Although EV71 co-localized with LAMP2, RAB9, and M6PR during its uncoating period, our results suggest that LAMP2 and RAB9 are not essential for EV71 replication. Based on these findings, amphisomes containing LAMP2, RAB9, and M6PR are not likely to be required for EV71 uncoating. Indeed, we could not find EV71 in organelles having double limiting membranes similar to those described for autophagosomes or amphisomes. Thus, EV71 is likely to localize to LEs, and not to amphisomes during the uncoating period (30 to 40 mai). At 40 mai, it was difficult to detect virus particles by immunoelectron microscopy. Accordingly, the number of viral antigens observed by immunofluorescence was highly reduced at 40 mai compared to that at 30 mai or earlier timepoints (data not shown). From these results, the compartments containing EV71 are predicted to undergo fusion with degradative organelles before or at 40 mai. Indeed, RAB9-positive organelles have been reported to fuse with compartments containing RAB7 (Stenmark, 2009), a process that might lead to the degradation of EV71 present in their lumen. (Kirkegaard, 2013). Furthermore, since hSCARB2 and LAMP2 target their binding proteins to lysosomal degradation (Blanz et al., 2010; Reczek et al., 2007; Cuervo and Dice, 1996; Ebato et al., 2008), it is likely that both hSCARB2 and LAMP2 direct EV71 to the degradative pathway. This may explain why we observe an apparent increase of EV71 RNA upon treatment with 3-MA, when organelles containing EV71 does not acquire LAMP2 (Fig. S2C). In the presence of 3-MA, 24% of the viral RNA co-localized with M6PR, in stark contrast to the 9.0% observed in the absence of 3-MA at 30 mai. However, we cannot rule out the possibility that overexpression of hSCARB2 may affect the dynamics of the virus in these cells and as such, influence some of our findings.

Since EV71 starts replication in the cytoplasm by IRES-mediated initiation of translation (Thompson and Sarnow, 2003), it is likely that the entry of genomic viral RNA in the cytoplasm leads to productive infection. On the other hand, the viral RNA present in the lumen of LEs would be exposed to a degradative environment, leading to abortive infection (Fig. S3). In the case the uncoating of the virus occurs on ILVs in LEs, viral RNA is released into LE lumen, whereas when the uncoating occurs on LE membrane, viral RNA is released into cytoplasm (Dang et al., 2014). As shown in Fig. S2C, we observed more EV71 RNA in the presence of 3-MA than in the absence of 3-MA, whereas 3-MA inhibited virus productive uncoating (Fig. S2A). These results suggest that some of the EV71 uncoating occur on ILVs in LEs and 3-MA inhibits uncoated EV71 RNA to leave LEs at 30 mai. Thus, it is likely that 3-MA increases abortive infection of EV71. According to a previous report (Lee et al., 2014), 3-MA reduced the replication and pathogenesis of EV71 (strain 4643) in a suckling mouse model. In 16HBE cells, 10 mM 3-MA inhibited the replication of EV71 (sub-genotype C4, GenBank: EU812515.1) (Song et al., 2018). Our strategy utilizing RD-hSCARB2 cells and light sensitive virus allowed us to analyze viral uncoating and replication separately. As a result, we found that 5 mM 3-MA did not affect the productive uncoating of EV71 and did not have a significant effect on viral replication (Fig. S2A).

The pH of the immature LEs is around pH 6.0, whereas mature LEs enriched in BMP-containing ILVs is pH 5.5 or lower (Nour and Modis, 2014). EV71 bound to hSCARB2 significantly starts uncoating at pH 6.0 or below *in vitro* (Yamayoshi et al., 2013). At pH 6.5, 2.6% of the virus was uncoated after 1 h, whereas this percentage raises to 5.0% and 6.1% at pH 6.0 or pH 5.5, respectively. From these results, only cellular compartments with a pH of 6.0 or lower would be compatible with viral uncoating. However, in the case of poliovirus (PV), which requires solely its receptor for uncoating, about 90% of the virus particles lose their coat 1 h after addition of its receptor (Arita et al., 1998). Thus, the efficiency for uncoating of EV71 bound to hSCARB2 at pH 6.0 is likely to be much lower than that of PV. This difference might imply that other factors assist EV71 uncoating besides hSCARB2 and the low pH.

Our results suggest that CD-M6PR is essential for EV71 replication, including uncoating. CD-M6PR is enriched in ILVs (Dikic, 2006). Both CI-M6PR and CD-M6PR have M6P-binding sites, and CI-M6PR interacts with hSCARB2 holding β-GC via M6P in cis (Zhao et al., 2014). Since CD-M6PR has a short extracellular domain and hSCARB2 is modified with M6P at the distal site from the membrane, CD-M6PR might interact with M6P on hSCARB2 in trans. The hypothesized interaction between CD-M6PR and hSCARB2 in trans would facilitate the binding between EV71 with another hSCARB2 molecule. It is possible that the action of multiple hSCARB2 molecules localized on both LEs and the ILVs would enhance the uncoating kinetics of EV71. However, future work will be necessary to validate this hypothesis. Since knockdown of M6PR may influence the distribution of SCARB2, we cannot exclude the possibility that M6PR interferes indirectly with the uncoating of the virus.

Efficient uncoating of EV71 occurs at mature LEs containing ILVs. Both immature and mature LEs have a pH low enough for EV71 uncoating; however, EV71 is not uncoated efficiently in immature LEs. A possible reason for this might reside in the structural difference between immature and mature LEs. In immature LEs, which harbor fewer ILVs, EV71 would be mainly localized mainly on the delimiting LE membrane, since uncoating of the virus occurs solely on membranes. In contrast, in mature LEs, both EV71 and hSCARB2 would localize on LE and ILV membranes, thus increasing the potential for uncoating. An alternative, yet not exclusive possibility is linked to the pH dependent interaction of hSCARB2 with β-GC. Previous *in vitro* studies demonstrated that hSCARB2 binds β-GC at pH 6.5, whereas it does not at pH 5.5 (Zhao et al., 2014). Interestingly, the binding site(s) of hSCARB2 for β-GC [amino acids 145-222 (Zachos et al., 2012) or 150–167 (Reczek et al., 2007)] overlaps with the binding site(s) for EV71 [amino acids 142-204 (Yamayoshi and Koike, 2011) or 144–151 (Chen et al., 2012)], and exogenous β-GC has been found to interfere with EV71 binding to hSCARB2 on the cell surface (Nakata et al., 2017). This implies that release of β-GC from hSCARB2 at pH 5.5 may enhance the interaction between EV71 and hSCARB2, thus boosting EV71 uncoating.

## MATERIALS AND METHODS

### Cells

RD cells and Vero cells were cultured in Dulbecco's modified Eagle's medium (Nissui) supplemented with 5% fetal bovine serum (FBS) and penicillin-streptomycin (Life Technologies) (5% FBS-DMEM). RD-hSCARB2 cells (generously gifted by Dr. K. Fujii) were cultured in 5% FBS-DMEM supplemented with 4 μg/ml puromycin (Calbiochem). Cells had been tested for contamination and mycoplasma infection.

### Viruses

The EV71 strains SK-EV006/Malaysia/97 (SK-EV006; genogroup B) (Shimizu et al., 1999), EV71/86-11316 NETH-86 (genogroup B) (Nakajima et al., 2012) and EV71-GFP (generously gifted by Dr. M. Arita), which expresses GFP upon viral replication (Yamayoshi et al., 2013), were used in this study. EV71/86-11316 NETH-86 was used for producing light-sensitive virus, and EV71-GFP was used in siRNA knockdown experiment. Unless otherwise stated, we used purified EV71 SK-EV006 as a representative strain of EV71. Experiments using recombinant DNA and pathogens were approved by the Committee for Experiments using Recombinant DNA and Pathogens at the Tokyo Metropolitan Institute of Medical Science (TMiMS).

### Antibodies

We used the following primary antibodies for immunofluorescence and western blotting: anti-SCARB2 goat antibodies (1:1250, AF1966, R&D Systems), anti-EEA1 rabbit antibodies (1:500, ab2900, Abcam), anti-clathrin heavy chain rabbit antibodies (1:1000, ab21679, Abcam), anti-RAB5 rabbit antibodies (1:1000, ab18211, Abcam), anti-RAB7 rabbit monoclonal antibody [EPR7589] (1:250 for immunofluorescence, 1:1000 for western blotting, ab137029, Abcam), anti-RAb9 mouse monoclonal antibody [EPR13272] (1:100 for immunofluorescence, 1:1000 for western blotting, ab179815, Abcam), anti-LAMP1 rabbit antibodies (1:1000, ab24170, Abcam), purified anti-LBPA mouse antibody (6C4) for BMP staining (1:500, Z-PLBPA, Echelon), anti-LAMP2 [EPR13272] rabbit monoclonal antibody (1:100, ab37024, Abcam), anti-EV71 mouse monoclonal antibody N3 (1:9000, from G. Liu and P. C. Choi-Sing), anti-EV71 serum (1:1000 for immunofluorescence, 1:200 for immunoelectron microscopy, kindly provided by H. Shimizu, NIID, Japan) (Nagata et al., 2002), anti-β-actin AC74 monoclonal antibody (1:5000, A5316, Sigma-Aldrich). For anti-M6PR antibodies, we used anti-CI-M6PR/IGF2R rabbit monoclonal antibody [EPR6599] (1:250, ab124767, Abcam), anti-CD-M6PR rabbit polyclonal antibodies (1:50, ABIN357957, Antibodies-online) and chicken polyclonal antibodies (1:200, GW21444, Sigma-Aldrich) for immunofluorescence, and anti-CI-M6PR/IGF2R rabbit antibody [EPR6599] (1:50,000, ab124767, Abcam), anti-CD-M6PR chicken polyclonal antibodies (1:500, GW21444, Sigma-Aldrich) for western blotting. As secondary antibodies, we used AlexaFluor488, 568, and 647 donkey, or goat anti-mouse, rabbit, chicken or goat IgG (H+L) (1:1000, Life Technologies), anti-IgG (H+L), 5 nm gold-conjugated goat anti-rabbit IgG (H+L), EM (1:100, EM.GARHL5, BBI Solutions), anti-rabbit IgG HRP, and anti-mouse IgG HRP (1:100,000, Jackson ImmunoResearch).

### Virus purification

RD-hSCARB2 cells were infected with EV71 SK-EV006/Malaysia/97 strain at an MOI of 5. Cells and media were frozen 18 h post-infection. After thawing, cell debris was removed by centrifugation in a NA-4HS rotor (TOMY) at 10,000 rpm for 20 min at 4°C. The supernatant was layered onto a 1.25 g/ml and 1.48 g/ml CsCl discontinuous step gradient in phosphate-buffered saline [PBS (−)] (per liter, 8.00 g NaCl, 1.15 g Na_2_HPO_4_, 0.20 g KCl, 0.20 g KH_2_PO_4_ [pH 7.4]). Native virions (F-particles) were collected from a fraction between the 1.25 g/ml and 1.48 g/ml CsCl. The F-particles were applied onto PD-10 or NAP5 column and eluted with PBS (−). After fractionation (0.2 ml/fraction), the fractions that included the F-particles were collected.

### Light-sensitive EV71

The experiments were performed in the dark unless stated otherwise. RD-hSCARB2 cells were infected with EV71/86-11316 NETH-86 strain at an MOI of 0.1 in 10 ml of 5% FBS-DMEM and incubated at 37°C for 1 h. Upon removal of the supernatant, cells were washed with DMEM and then 20 ml of 5% FBS-DMEM and 40 µg/ml neutral red were added. Around 24 h after infection, the infected cells were transferred at −80°C followed by freezing and thawing three times. After a sonication for 30 min on ice, the virus solution was centrifuged, and the supernatant was recovered followed by titration in the dark or after light irradiation using TCID_50_ in VERO cells. RD-hSCARB2 was infected with the recovered virus similarly to the first stage infection. The virus supernatant was recovered followed by titration using TCID_50_ in VERO cells and used for experiments. For light irradiation of the light-sensitive virus, light was shone 30 cm apart from the plates by Grassy LeDio RS122 Fresh White LED light (Volxjapan) including 450 nm spectrum peak for 30 min at room temperature. The temperature of the plates was strictly monitored to avoid cell overheating. In experiments described in Fig. 1A and 1B, RD-hSCARB2 cells were infected with light-sensitive EV71 at a MOI of 10 and cells were either kept in the dark or irradiated at the indicated time after infection to inactivate intact virus. Experiments were performed in triplicate. Seven hours after infection, cells were harvested, and the viral titers were determined using TCID_50_ in VERO cells. In the experiments shown Fig. S2A, RD-hSCARB2 cells were infected with light-sensitive EV71 at an MOI of 0.004 in the presence or absence of 5 mM 3-MA. Cells were either kept in the dark or irradiated with light to inactivate the intact virus at 10 or 40 mai. After irradiation, the medium containing 3-MA was replaced with fresh medium in selected samples to assess the inhibitory effect of 3-MA on viral replication. These experiments were performed in triplicate. Twenty-three hours after infection, cells were harvested, and the viral titers determined using TCID_50_ in VERO cells.

### Immunofluorescence

RD-SCARB2 cells and RD cells were seeded onto 16-well Lab-Tek Chamber Slide (Nunc). One day after seeding, purified EV71 were added at an MOI of 25 at 4°C to allow viral attachment to the cell surface without entry. In selected cases, cells were pretreated with or without 40 mM NH_4_Cl or 5 mM 3-MA for 30 min. Cells were then shifted to 37°C (designated as time 0), washed with PBS (-) with or without NH_4_Cl or 3-MA, fixed in PBS containing 4% paraformaldehyde (PFA) for 15 min and washed four times with cold PBS before further processing. In Fig. 3, to detect EGF localization, we added 2 μg/ml of biotinylated EGF bound to AlexaFluor555 streptavidin (Invitrogen) in the medium. Fixed cells were incubated with PBS containing 0.05% saponin and 5% bovine serum albumin fraction V (Sigma-Aldrich) to permeabilize the membrane and block nonspecific reactions. Samples were incubated overnight at 4°C with primary antibodies. After being washed with PBS (-), samples were then incubated with the secondary antibodies for 90 min at room temperature, and after another PBS (-) wash, mounted in Vectashield with DAPI Mounting Medium (Vector Laboratories). Samples were imaged with a laser-scanning microscope (TCS SP2, Leica Microsystems). Images were analyzed by Imaris (Zeiss).

### *In situ* immunofluorescence analysis

Cells were pretreated with or without 40 mM NH_4_Cl or 5 mM 3-MA for 30 min followed by addition of purified EV71 at an MOI of 25 with or without NH_4_Cl or 3-MA. Infected cells were washed twice with PBS (-) with or without NH_4_Cl or 3-MA followed by fixation with 4% PFA at room temperature for 30 min. Fixed cells were washed with PBS (-) three times, dehydrated with ethanol and transferred at −20°C. After rehydration, samples were treated with proteases and *in situ* hybridization was performed using QuantiGene ViewRNA ISH (Thermo Fisher Scientific) according to the manufacturer's instructions followed by fluorescence analysis. EV71 sequence was from nucleotide number 3008 to 3609 of SK-EV006/Malaysia/97 strain (GenBank: AB469182.1) (Shimizu et al., 1999).

### EV71 infection in knockdown cells

RD cells were seeded in eight-well culture slides (BD Falcon) 1 day before transfection with siRNAs. siRNAs, pre-designed or validated *Silencer* Select siRNAs were obtained from Life Technologies. As a negative control, *Silencer* Negative Control No. 1 siRNA (Life Technologies) was used. Transfection of siRNAs into RD cells was performed using Lipofectamine 3000 (Invitrogen, Carlsbad, CA, USA), which was diluted in Opti-MEM and added to siRNA diluted in Opti-MEM. After an incubation of 5 min at room temperature, the mixture was added to cells and incubated at 37°C for 4 h followed by medium exchange. Two days later, cells were infected with EV71-GFP at an MOI of 0.5. Experiments were performed in triplicate for knockdown samples and sextuplicate for siControl. In parallel, cells in other wells were fixed in PBS (-) containing 4% PFA for 30 min followed by immunostaining of target proteins to check the knockdown efficiency. Immunostaining showed knockdown efficiency consistent with the results obtaining using western blotting. After 24 h incubation at 37°C, infected cells were fixed in PBS containing 4% PFA for 30 min and washed four times with PBS. The numbers of GFP-positive cells were counted and the ratio against the numbers of DAPI-positive cells was analyzed. GFP positive cells were observed by VS120 Virtual Slide Microscope (Olympus) and analyzed using ImageJ software (National Institutes of Health, USA). To analyze protein expression by immunofluorescence, cells were stained as described above and imaged with a laser-scanning microscope. To quantify protein expression, samples were loaded onto 12% Mini-Protean TGX precast gels (Bio-Rad), followed by western blotting with anti-RAB5, RAB7, RAB9, M6PRs, and clathrin heavy chain antibodies. RD cells treated with siRNAs were detached with 0.02% EDTA-PBS and dissolved in 100 μl of electrophoresis sample buffer (50 mM Tris-HCl, pH 6.8, 10% glycerol, 2% sodium dodecyl sulfate, 0.1% bromophenol blue in H_2_O) per 2×10^5^ cells, followed by sonication and addition of 5% v/v of 2-mercaptoethanol. Samples were loaded onto precast gels mini-Protein TGX Gels 12% (Bio-Rad), followed by western blotting. Band intensities were analyzed using ImageJ.

### Electron microscopy

For transmission electron microscopy, cells washed with PBS (-) were fixed in 2% (w/v) PFA and 2.5% (v/v) glutaraldehyde in PBS (-) for 2.5 h. After osmication in 1% (w/v) osmium tetroxide, specimens were dehydrated through a graded alcohol series, and embedded in Epon 812 (TAAB Laboratories). Ultrathin sections were cut on an Ultracut microtome (Leica EM-UC6) and stained with uranyl acetate and lead citrate. Sections were observed under a transmission electron microscope (Hitachi H-7650).

For immuno-electron microscopy, cells washed with PBS (-) were fixed in 4% PFA and 0.05% glutaraldehyde in PBS (-) for 2.5 h. The immunogold labeling followed the method of Akagi et al. (Akagi et al., 2006), except for the antibodies. After blocking with 10% normal goat serum, sections were incubated in Tris-buffered saline (TBS) containing antibody against EV71 for 24–48 h at 4°C and then washed with TBS and incubated with gold particle-conjugated secondary antibodies for 2 h at room temperature. Thereafter, the sections were rinsed and embedded with a mixture of 1% polyvinyl alcohol containing 0.1% uranyl acetate, dried, and observed with an electron microscope (Hitachi H-7650).

### Statistical analysis

Statistical analysis was done using GraphPad Prism 7.00 (GraphPad Software Inc.). A two-tailed unpaired *t*-test has been performed in Figs 1D, 3B, 6B, 7B, and S2A, and a two-tailed Fisher's exact test has been performed in Figs S1C and S2C.
